# mSphere of Influence: Seeking the unseen fungi in tumors

**DOI:** 10.1128/msphere.00988-24

**Published:** 2025-01-28

**Authors:** Ning-Ning Liu

**Affiliations:** 1 State Key Laboratory of Systems Medicine for Cancer, Center for Single-Cell Omics, School of Public Health, Shanghai Jiao Tong University School of Medicine, Shanghai, China; Shenzhen Institute of Synthetic Biology, Chinese Academy of Sciences, Shenzhen, China

**Keywords:** fungi, cancer, fungi and cancer, fungal-host interaction

## Abstract

Ningning Liu works in the field of fungal infection and cancer progression, with a particular focus on the mechanism of host-pathogen interaction. In this mSphere of influence article, he reflects on how papers entitled “The fungal mycobiome promotes pancreatic oncogenesis via activation of MBL,” by B. Aykut, S. Pushalkar, R. Chen, Q. Li, et al. (Nature 574:264–267, 2019, https://doi.org/10.1038/s41586-019-1608-2), and “A pan-cancer mycobiome analysis reveals fungal involvement in gastrointestinal and lung tumors,” by A. B. Dohlman, J. Klug, M. Mesko, I. H. Gao, et al. (Cell 185:3807–3822.E12, 2022, https://doi.org/10.1016/j.cell.2022.09.015), emphasized the non-negligible role of fungi in the host and demonstrated a connection between fungi and cancer. These researches arouse his interest in seeking the novel fungal pathogen lurking inside tumors and understanding the unexplored mechanisms behind the severe fungal infections in cancer patients.

## COMMENTARY

According to the latest clinical statistics, the number of cancer cases attributed to infections accounts for about 13% of cancer patients worldwide each year. Meanwhile, in China, nearly 30% of cancer patients die due to infections, with the main pathogens including *Helicobacter pylori*, human papillomavirus, hepatitis B virus, and *Clonorchis sinensis* (1). However, as a critical component of the human microbiome and frequently isolated pathogens in clinics, the role of fungi in tumorigenesis and progression remains unclear. Currently, about 1 billion people worldwide suffer from fungal infections each year, resulting in 3.75 million deaths. Among them, cancer patients are the most susceptible populations to fungal infections, with a mortality rate of 40%–78% (2–5). The papers “The fungal mycobiome promotes pancreatic oncogenesis via activation of MBL” (6) and “A pan-cancer mycobiome analysis reveals fungal involvement in gastrointestinal and lung tumors” (7) emphasized the role of fungi in cancer and prove the inextricable link between fungi and cancer progression.

In “The fungal mycobiome promotes pancreatic oncogenesis via activation of MBL,” the authors discovered that the fungal load in pancreatic cancer (PDA) patients is approximately 3,000 times higher than in healthy controls. The composition of fungi in PDA is markedly distinct from that in the pancreas or gut of healthy individuals, with a notable enrichment of fungi from the genus *Malassezia* in PDA tissues. Experimental evidence demonstrated that *Malassezia* promotes PDA progression by activating the mannose-binding lectin (MBL) pathway, which triggers the complement cascade (C3). Furthermore, the study suggests that fungi can migrate from the gut to the pancreas via the gut-pancreas axis, altering the pancreatic microenvironment and facilitating tumor development.

The association between fungi and PDA highlighted in this research offers potential new therapeutic targets, such as antifungal treatments or interventions to block the MBL-C3 axis, for managing PDA. In addition, fungal communities may serve as biomarkers for early diagnosis or disease monitoring. This study is the first to elucidate the pathogenic role of fungi in the initiation and progression of PDA, underscoring the potential value of fungal-targeted therapeutic strategies.

In “A pan-cancer mycobiome analysis reveals fungal involvement in gastrointestinal and lung tumors,” the authors systematically analyzed the presence and characteristics of fungi across 35 types of cancers, revealing their specific distribution and potential impact in different cancer types. The study detected fungal DNA and fungal cells in various human cancers. Although with low relative abundance, the fungal community composition varied significantly among different cancers. The presence of certain fungi was found to be significantly associated with factors such as patient age, tumor subtype, smoking status, immune therapy response, and survival rates. This study represents the first comprehensive mapping of cancer-associated fungi, highlighting their potential role in cancer biology. Understanding the relationship between fungi and cancer could provide new perspectives on cancer diagnosis, treatment, and prognosis. For instance, the presence of specific fungi may serve as biomarkers for early detection or to predict therapeutic responses.

These papers made me wonder whether fungi are associated with other cancers and how fungi contribute to cancer progression. Thus, our team mainly focused on the tripartite interactions among microecology, fungi, and host, seeking the tumorigenic fungi and the specific gene targets to elucidate the molecular mechanisms of fungal-host interactions, aiming to provide novel therapeutic insights on fungal infections and cancer progression.
